# Irradiation with 365 nm and 405 nm wavelength shows differences in DNA damage of swine pancreatic islets

**DOI:** 10.1371/journal.pone.0235052

**Published:** 2020-06-25

**Authors:** M. Klak, M. Gomółka, T. Dobrzański, G. Tymicki, P. Cywoniuk, P. Kowalska, K. Kosowska, T. Bryniarski, A. Berman, A. Dobrzyń, J. Idaszek, W. Święszkowski, M. Wszoła

**Affiliations:** 1 Foundation of Research and Science Development, Warsaw, Poland; 2 Department of Biochemistry, Nencki Institute of Experimental Biology, Warsaw, Poland; 3 Faculty of Materials Science and Engineering, Warsaw University of Technology, Warsaw, Poland; Georgia Institute of Technology and Emory University, UNITED STATES

## Abstract

**Introduction:**

3D printing is being used more extensively in modern biomedicine. One of the problems is selecting a proper crosslinking method of bioprinted material. Amongst currently used techniques we can distinguish: physical crosslinking (e.g. Ca^2+^ and Sr^2+^) and chemical crosslinking–the UV light crosslinking causing the biggest discussion. UV radiation is selectively absorbed by DNA, mainly in the UV-B region but also (to some extent) in UV-A and UV-C regions. DNA excitement results in typical photoproducts. The amount of strand breaks may vary depending on the period of exposition, it can also differ when cells undergo incubation after radiation.

**Aim:**

The aim of this study was to show whether and how the time of irradiation with 405 nm and 365 nm wavelengths affect DNA damage in cell lines and micro-organs (pancreatic islets).

**Materials and methods:**

The degree of DNA damage caused by different wavelengths of radiation (405 nm and 365 nm) was evaluated by a comet assay. The test was performed on fibroblasts, alpha cells, beta cells and porcine pancreatic islets after 24 hours incubation period. Samples without radiation treatment were selected as a control group. Results analysis consisted of determining the percent of cells with damaged DNA and the tail intensity evaluation.

**Results:**

The degree of DNA damage in pancreatic islets after exposure to 405 nm wavelength oscillated between 2% and 6% depending on the tested time period (10 – 300 seconds). However, treating islets using 365 nm wavelength resulted in damage up to 50%. This clearly shows significantly less damage when using 405 nm wavelength. Similar results were obtained for the tested cell lines.

**Conclusions:**

Crosslinking with 405 nm is better for pancreatic islets than crosslinking with 365 nm UV light.

## Introduction

Creating bionic organs is currently the main focus of many research facilities around the world. 3D-bioprinting of organs is the future of transplantation as it is the solution to many significant problems, such as donor shortage or immune response. Nevertheless, this is still a very complex and complicated process. [1, 2]. There are four major factors of bioprinting that play a crucial role in the process: bioink, printing process, construct geometry and cell viability [3].

One of the most important steps in the bioprinting procedure is choosing the right crosslinking method of the bioprinted material. The reason for this step is maintaining the correct physical form and good shape of bioprinted construct that will allow its implementation in the future [4]. It can be achieved using a few different techniques. Amongst those are: physical crosslinking (Ca^2+^ or Sr^2+^), chemical crosslinking (including thermal and UV crosslinking) [5]. To produce bionic organs, such as pancreas, liver or heart, researchers must use cell-laden bioinks. UV light can be a good crosslinking agent while printing without cells but it can be harmful when a decision to implement cell-laden bioink is made. This is because UV light is considered to be a DNA damaging agent [6,7].

UV light can be divided into three categories: UV-A (wavelength– 315-400nm), UV-B (wavelength– 280-315nm), UV-C (wavelength– 200-280nm). UV radiation, especially UV-B (UV-A and UV-C to some extent), is selectively absorbed by DNA, which may induce typical photoproducts. The most abundant DNA lesions (mutagenic and cytotoxic) resulting from UV radiation are pyrimidine dimers (so called CPDs = cyclobutene pyrimidine dimers) and 6–4 photoproducts (6–4 PPs). Respectively, CPDs and 6-4PPs make up about 75% and 25% of all the UV-resulting DNA damage [8, 9]. Additional damage in the form of single-strand breaks and double-strand breaks might also occur [10]. There is some evidence indicating that the incubation period after the UV light exposition is a reason for a varying amount of total DNA damage in cells, suggesting that DNA repair takes place during that period [11]. At the moment there are a few DNA repair mechanisms known and studied, which we can divide into: photoreactivation, excision repair (BER–base excision repair, NER–nucleotide excision repair, MMR–mismatch repair), mutagenic repair or lesion bypass and recombinational repair. Photoreactivation process involves a light-dependent enzymatic reaction (using photolyase enzyme) that results in monomerization of dimerized pyrimidines (usually thymine residues). All types of excision repair depend on cutting out (splicing out) the UV-treated region and replacing it with non-damaged bases. Mutagenic repair and lesion bypass employ “sloppy copiers” which help to replicate DNA with non-complimentary bases. Finally, recombinational repair restores both double-strand and single-strand breaks through either DSB repair (double-strand break repair) or NHEJ (non-homologous end joining) [8,12].

It is commonly known that UV light wave 365 nm may cause reversible and irreversible damage to all types of cells, with distinction depending on time period of exposure to radiation and incubation after radiation. On the other hand, the scientific literature offers are few results indicating the influence of a wavelength of 405 nm on a cell's DNA. 405 nm wavelength is a novel crosslinking agent also used during bioprinting process. It is considered a safer method of stabilizing bioprinted material, with a wavelength being just above the UV light range, already in the visible light [13].

The purpose of this study is to show first insights into supposed DNA damage of pancreatic islets, α-cells and β-cells when treated with 405 nm irradiation, compared to 365 nm crosslinking which can also be used in this procedure. This will be achieved through performing the comet assay procedure.

## Materials

### Pancreatic islets

Porcine pancreas was digested with collagenase NB8 (Nordmark, S1745602) and then was cultured for 24 h in CMRL 1066 (Gibco, 21530–027) with 10% FBS (EUR X Molecular Biology Products, E5050-03), 100 IU/mL penicillin and 100 μg/mL streptomycin (Corning, 30-002-Cl) and 5 mM glucose (Sigma Aldrich, G8270), in 37°C and 5% CO_2_.

Three cell lines were used for the study.

### Alpha cells

αTC1.6—alphaTC1 Clone 6—alpha cell from pancreas of the Mus musculus diseased on adenoma. This cell line was a gift from A. Dobrzyń, Nencki Institute of Experimental Biology, Polish Academy of Sciences, Warsaw, Poland.

Cell cultivation was performed in DMEM, Low Glucose, Pyruvate (Gibco, 11885–084) supplemented with 100 IU/mL penicillin and 100 μg/mL streptomycin (Corning, 30-002-Cl), 25 μg/mL amphotericin B (Corning, 30-003-CF), 10% FBS (EUR X Molecular Biology Products, E5050-03), 0.02% BSA (Sigma-Aldrich, A7906), 15 mM HEPES (Serva, 25247.02), 0.1 mM 1x MEM Non‐Essential Amino Acids (Gibco, 11140–035;) and 2 g/L D-glucose (Sigma Aldrich, G8270). Passages used were between no. 10 and 20. Incubation: 37°C and 5% CO^2^ atmosphere in New Brunswick Galaxy 170R incubator.

### Beta cells

INS-1E cells—rat insulinoma. This cell line was a gift from A. Dobrzyń, Nencki Institute of Experimental Biology, Polish Academy of Sciences, Warsaw, Poland.

Cell cultivation was performed in RPMI-1640 medium (Sigma R0883) supplemented with 100 IU/mL penicillin and 100 μg/mL streptomycin (Corning, 30-002-Cl), 25 μg/mL amphotericin B (Corning, 30-003-CF), 5% heat inactivated fetal bovine serum (FBS) (EUR X Molecular Biology Products, E5050-03), 2 mM L-glutamine (ScienCell, 0813), 10 mM 4-(2-hydroxyethyl)-1-piperazineethanesulfonate (HEPES) (Serva, 25247.02), 50 μM 2-mercaptoethanol (Sigma-Aldrich, M6250) and 1 mM sodium pyruvate (Serva, 15220.04). Passages used were between no. 80 and 90. Incubation: 37°C and 5% CO^2^ atmosphere in New Brunswick Galaxy 170R incubator.

### Fibroblasts

HFF1—Human Foreskin Fibroblasts. This cell line was acquired from ATCC, product number ATCC® SCRC-1041™.

Cell cultivation was performed in DMEM, Low Glucose, Pyruvate (Gibco, 11885–084) that was supplemented with 100 IU/mL penicillin and 100 μg/mL streptomycin (Corning, 30-002-Cl), 2.5 μg/mL amphotericin B (Corning, 30-003-CF), 20% FBS (EUR X Molecular Biology Products, E5050-03), 2 mM L-glutamine (ScienCell, 0813) and 2 g/L Dglucose (Sigma Aldrich, G8270). Passages used were under no. 10. Incubation: 37°C and 5% CO^2^ atmosphere in New Brunswick Galaxy 170R incubator.

## Methods

### UV radiation

Porcine pancreatic islets used in the experiment were exposed to visible blue light radiation (405 nm) in the following time periods: 10s, 30s, 60s, 90s, 120s, 180s and 300s. All cell lines (alpha cells, beta cells and fibroblasts) were exposed to the same wavelength of visible blue light (405 nm) in the following time periods: 10s, 60s, 120s and 300s. As a comparison, we checked the effect of 365 nm wavelength of UV light using the same time periods. Pancreatic islets and cells without any radiation treatment were selected as a control group.

**Fig 1:**
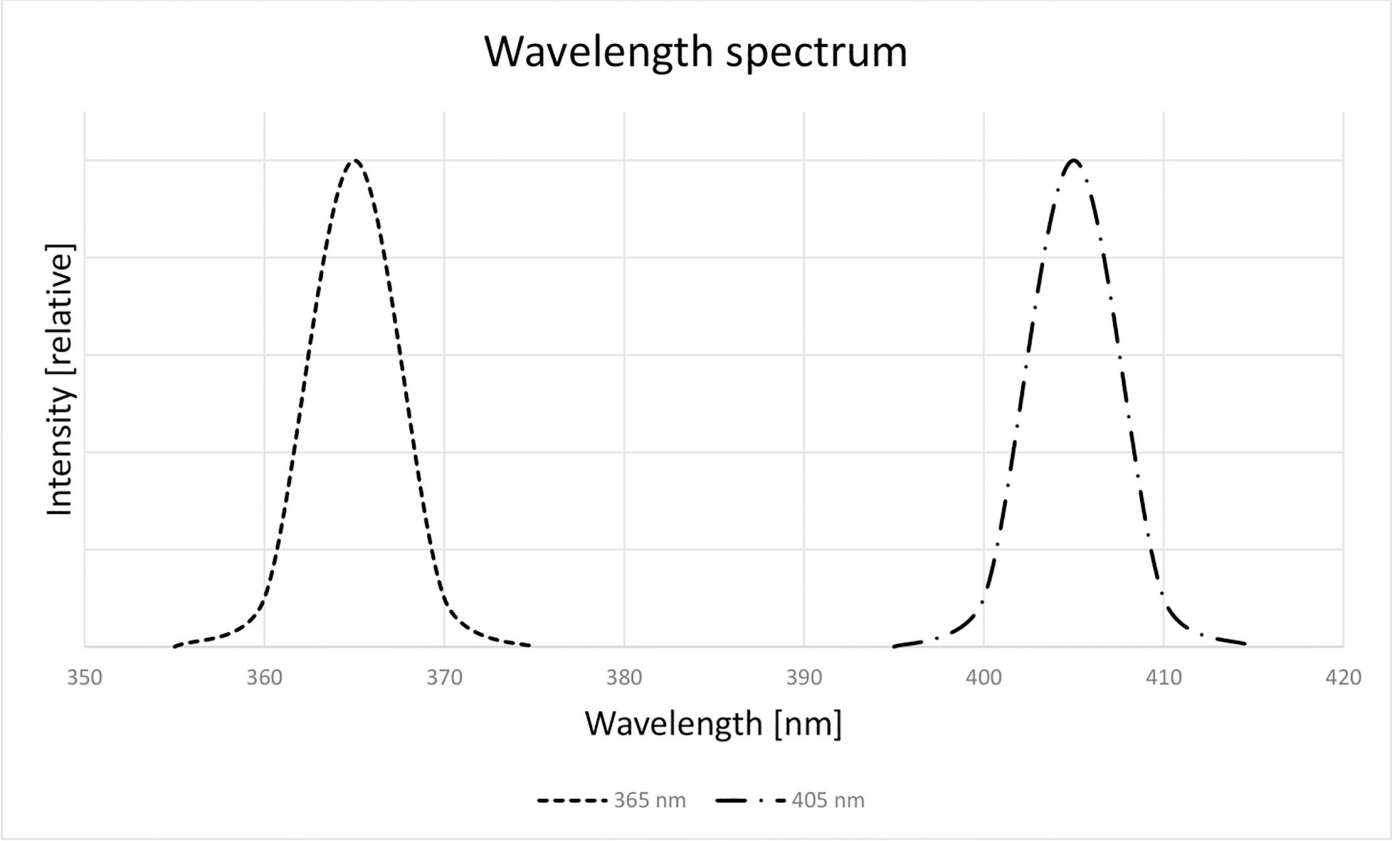
Spectra of devices used in the irradiation process. The device with typical peak wavelength 365 nm has its minimum at 360 nm and maximum at 370 nm. The device with typical peak wavelength 405 nm has its minimum at 400 nm and maximum at 410 nm.

Device parameters used in the irradiation process are presented in Table 1. Spectrum of both devices is shown in Fig 1. Irradiation was measured using an OmniCure R2000 Radiometer. A 3-fold measurement was made for each diode and the mean value was derived from the results obtained. Dose [J/m^2^] of radiation per sample (depending on radiation period and wavelength) is presented in Table 2. All samples were radiated in a Petri dish (5 cm diameter). Light source was placed 5cm above the Petri dish.

**Table 1:** Parameters of irradiation device used in this study.

Product no.	Irradiance [W/m^2^]	Min peak wavelength [nm]	Mean peak wavelength [nm]	Max peak wavelength [nm]
OSV1YL5111A	127,7	360	365	370
OSV5DL5111A	457,7	400	405	410

**Table 2:** Dose of radiation [J/m^2^] to which studied samples were subjected.

Time [seconds]	Dose [J/m^2^] 365 nm wavelength	Dose [J/m^2^] 405 nm wavelength
10	1277	4577
30	3831	13731
60	7662	27462
90	11493	41193
120	15324	54924
180	22986	82386
300	38310	137310

### Comet assay

To verify that damage to the cell nucleus is caused by 405 nm and 365 nm wavelength, commercially available alkaline (pH 10.0) comet assay was performed (OxiSelect™ Comet Assay Kit, STA-351). The comet assay and observations were carried out 24 hours after radiation to give cells a chance to repair DNA damage and adhere to the plate’s surface (where applicable). After incubation period cell lines were treated with trypsin, centrifuged and suspended in PBS. Pancreatic islets were further digested with trypsin to obtain single cell suspension, then centrifuged and suspended in PBS. Cell suspensions were mixed with agarose and placed on slides pre-coated with agarose. Slides were incubated for an hour in Lysis Buffer. Next, slides were incubated in Alkaline Solution for 30 minutes. Subsequently, slides were subjected to single-cell gel electrophoresis, rinsed with water and incubated in 70% ethanol and then left to dry. Slides were stained with DNA dye for 15 minutes. The analysis of the results consisted of the percentage of cells with damaged DNA (200 hundred cells for each sample were counted) and the tail size/intensity visual assessment.

The visual assessment was performed by means of marking comets in a scale from 0 to 4, with a total amount of 100 comets per sample [14]. The final score is a mean of individual scores in a sample, depending on the number of cells counted. The legend of this marking system is shown in Table 3.

**Table 3:** Size/intensity visual estimation of comets in cells of pancreatic islets treated with UV 365 nm wavelength, in alpha cells treated with UV light, in beta cells treated with UV light. Rating is the sum of every cell’s individual score divided by the number of cells counted. There were a 100 cells counted for each sample.

**Pancreatic islets**	**Rating**	**Alpha cells**	**Rating**	**Beta cells**	**Rating**	**Fibroblasts**	**Rating**
Control	0	Control	1	Control	1	Control	0
10s	1	10s	1	10s	1	10s	1
30s	2	30s	-	30s	-	30s	-
60s	3	60s	1	60s	1	60s	2
90s	2	90s	-	90s	-	90s	-
120s	2	120s	2	120s	2	120s	1
180s	4	180s	-	180s	-	180s	-
300s	4	300s	2	300s	2	300s	2
**Individual score:**			
**0**	Tail absent		
**1**	Low intensity		
**2**	Low-to-medium intensity		
**3**	Medium-to-high intensity		
**4**	High intensity		

**Fig 2:**
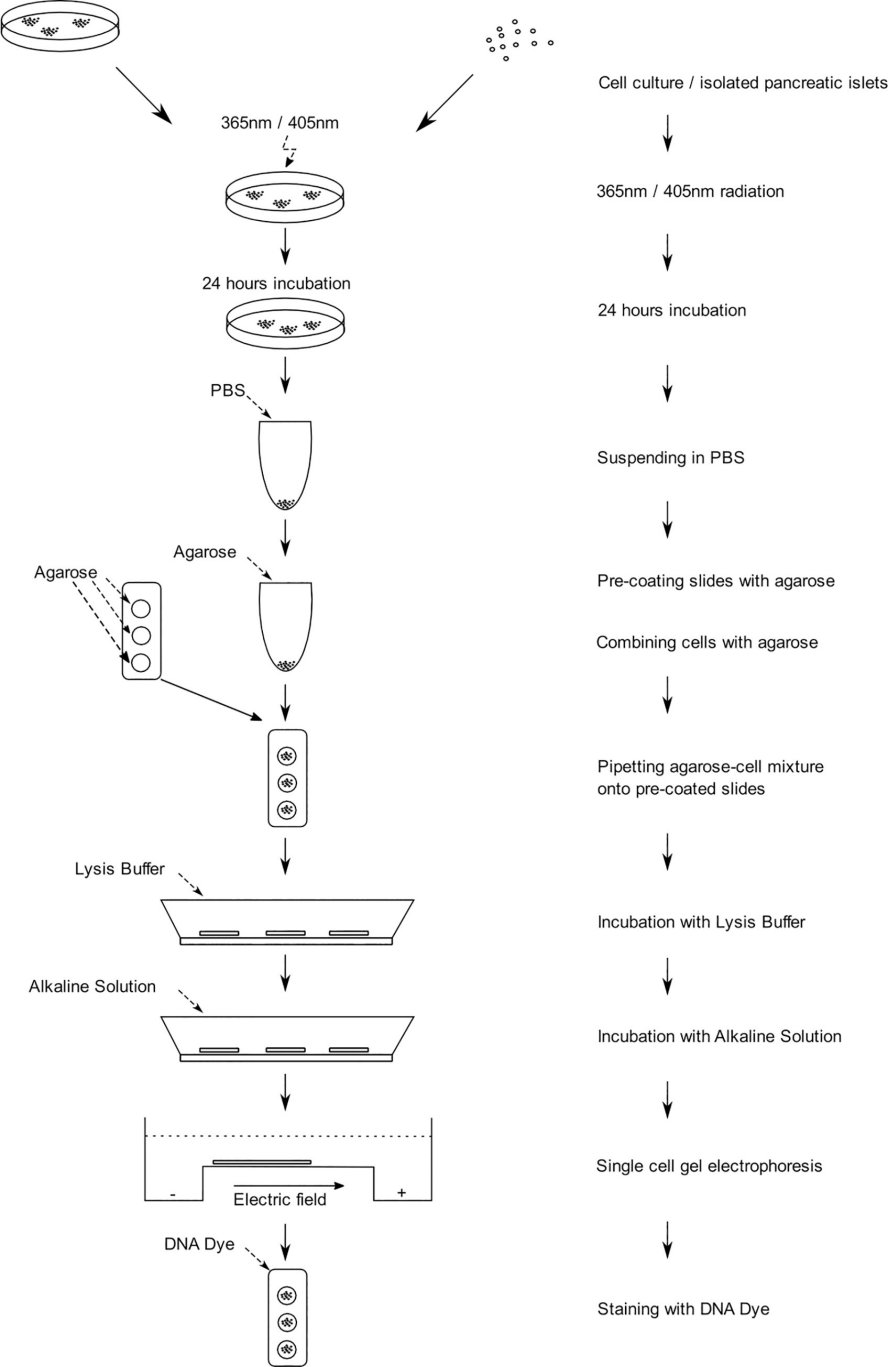
The diagram of steps that were performed during presented research based on the commercially available comet assay kit (Cell Biolabs, OxiSelect^TM^ Comet Assay Kit).

The diagram of all steps taken in this experimentation is presented in Fig 2.

### Statistical analysis

Statistical analysis of presented results was based on a calculated P-value. P-value was calculated by means of the Fisher’s exact test. P-values were accepted as statistically relevant when p < 0.05. Parameters where p-value was calculated are as follows: samples (pancreatic islets, alpha cells, beta cells, fibroblasts) that underwent radiation treatment (365 nm and 405 nm) compared to control (no radiation), samples (pancreatic islets, alpha cells, beta cells, fibroblasts) treated with 405 nm wavelength compared to samples treated with 365 nm wavelength.

## Results

### Pancreatic islets

**Fig 3:**
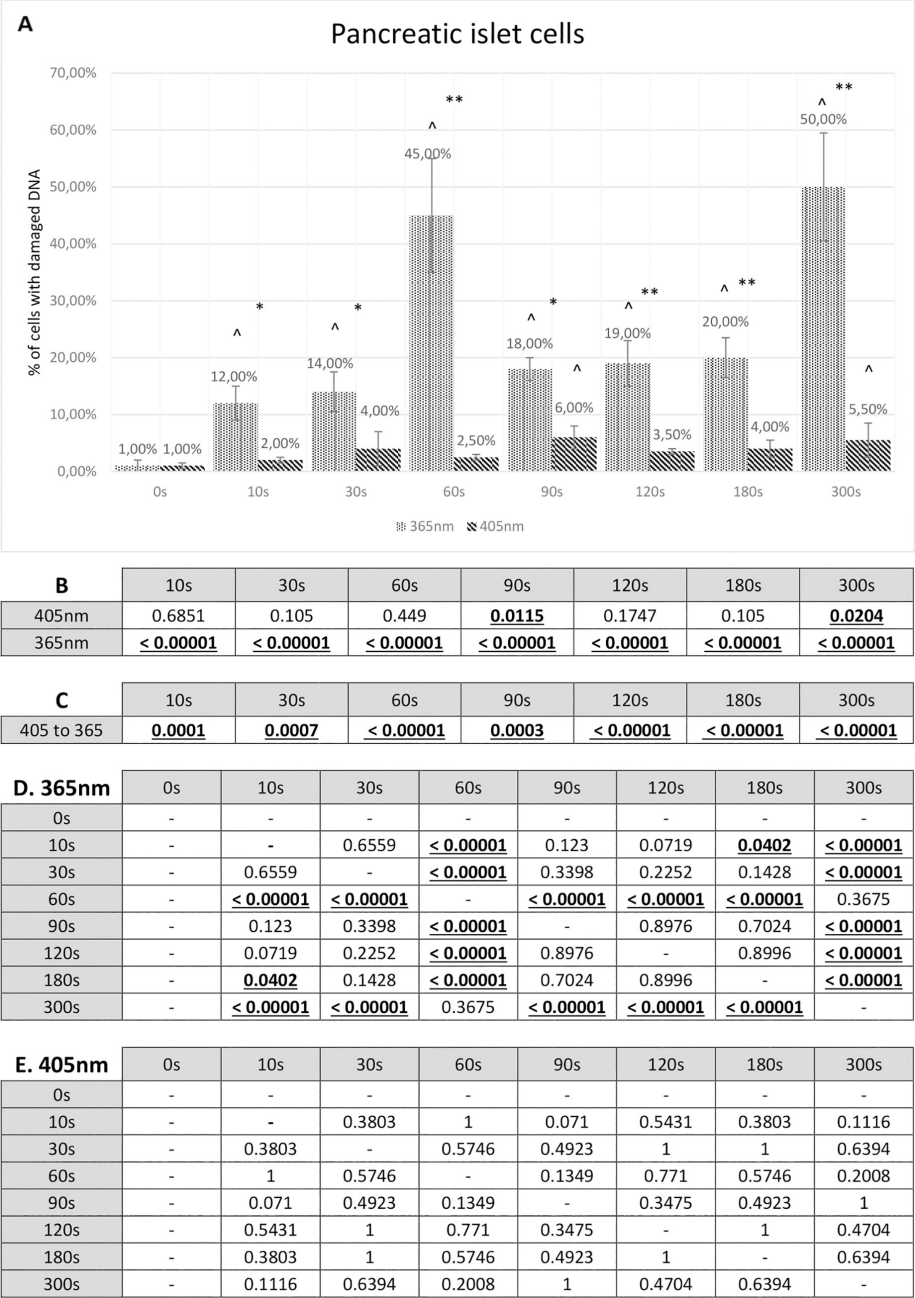
Comet assay results for pancreatic islets. **A** Comet assay results presenting the percentage of pancreatic islet cells with any degree of DNA damage. The Fisher exact test statistic value comparing 405 nm to 365 nm: * - p < 0.05; ** - p < 0.0001. The Fisher’s exact test statistic value comparing radiation (405 nm and 365 nm) to control: ^ - p < 0.05. Standard deviation bars are present for every value. **B** P-value calculated by means of the Fisher’s exact test. P-value of pancreatic islet cells used in the comet assay study compared to control. **C** P-value calculated by means of the Fisher’s exact test. P-value of pancreatic islet cells treated with 405 nm radiation compared to 365 nm radiation. Statistically relevant p-values, where p < 0.05, are shown underlined and in bold font. **D** P-value calculated by means of the Fisher’s exact test. P-values comparing different 365 nm radiation time periods. **E** P-value calculated by means of the Fisher’s exact test. P-values comparing different 405 nm radiation time periods.

Treating pancreatic islet cells with 405 nm wavelength for 120 seconds resulted in 3.5% cells with damaged DNA while 300 seconds of 405 nm radiation caused DNA damage in 5.5% of cells. Together with the rise of radiation time using wavelength 365 nm an increasing amount of damaged DNA was observed. 10 – 30 seconds of pancreatic islets radiation resulted in 12 – 14% damage of cell’s genetic material. 300 seconds of UV light treatment caused damage to 50% of cells. Interestingly, while treating pancreatic islets with 60 seconds of 365 nm wavelength, 45% of cells presented some damage in their nuclei DNA.Full results showing the percentage of pancreatic islet cells with damaged DNA are presented in Fig 3A.

What is more, a statistical analysis by means of the Fisher exact test was prepared and it shows P-values comparing 405 nm and 365 nm radiation. It is presented in the Fig 2A–2C. It has been calculated that all the values are statistically relevant, which is a prove of significant distinction between radiation with these two wavelengths. Four studied time periods reached P-value lower than 0.0001 (60 seconds, 120 seconds, 180 seconds and 300 seconds). Additionally, a statistical analysis by means of the same test was fixed to compare both wavelengths with the control. Radiation using 365 nm proved to have its P-value statistically relevant (less than 0.00001) while radiation using 405 nm had its P-value non-relevant in five samples (10 seconds, 30 seconds, 60 seconds, 120 seconds and 180 seconds) and relevant with a much higher result in the rest of the samples (none of them under 0.00001). This statistical analysis is shown in the Fig 3B. Furthermore, statistical analysis of the correlation between different time periods of radiation is presented in the Fig 3C and 3E.

**Fig 4:**
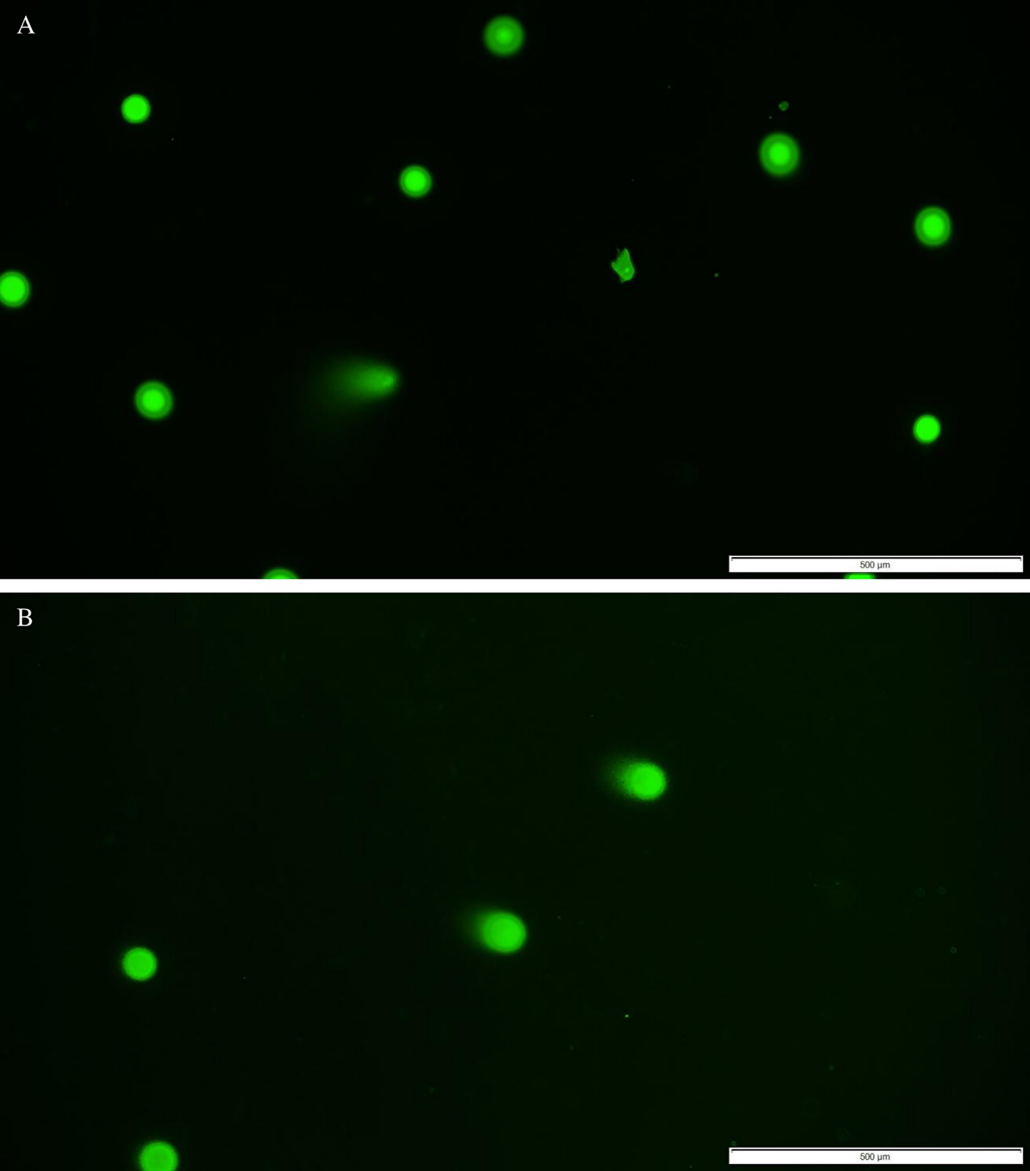
Examples of fluorescence microscopy photographs of comets in cells of pancreatic islets: **A** treated with UV 365 nm wavelength, magnification 10x; **B** treated with visible blue light 405 nm wavelength, magnification 10x.

However, the biggest difference was observed while analysing the intensity of comet tails in degenerated cells nuclei. The highest tail intensity was reached already after 180 seconds of treating with 365 nm wavelength. Low and low-to-medium tail intensity was represented in cells radiated through 10 – 120 seconds. Table 3 shows tail intensity estimation of 365 nm-treated pancreatic islets. Fig 4 presents photographs of the comet assay results taken using the fluorescence microscope.

### Alpha and beta cells

Cultured alpha and beta cells turned out to be more resistant to radiation than isolated pancreatic islets. Alpha cells subjected to visible blue light (405 nm) for 10 seconds resulted only in 1.5% overall DNA damage, while treating them with 365 nm wavelength (UV light) caused disturbance to 2% cells. The highest percentage of damaged alpha cells was observed in 300 s 365 nm wavelength-tested culture. It oscillated around 7.5%. The same time period and 405 nm wavelength led to only 2% damage.

**Fig 5:**
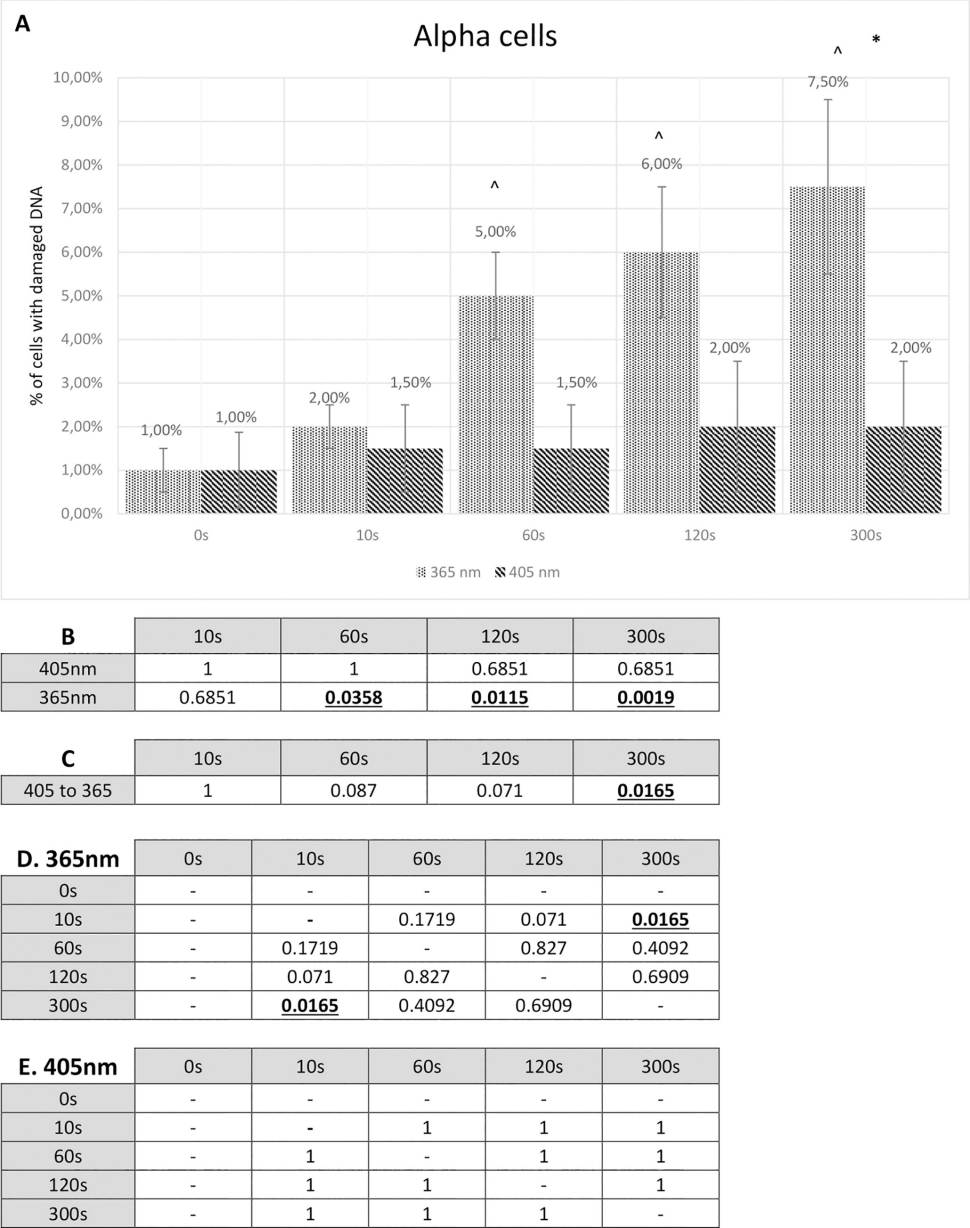
Comet assay results for alpha cells. **A** Comet assay results presenting the percentage of alpha cells with any degree of DNA damage. The Fisher exact test statistic value comparing 405 nm to 365 nm: * - p < 0.05; ** - p < 0.0001. The Fisher’s exact test statistic value comparing radiation (405 nm and 365 nm) to control: ^ - p < 0.05. Standard deviation bars are present for every value. **B** P-value calculated by means of the Fisher’s exact test. P-value of alpha cells used in the comet assay study compared to control. **C** P-value calculated by means of the Fisher’s exact test. P-value of alpha cells treated with 405 nm radiation compared to 365 nm radiation. Statistically relevant p-values, where p < 0.05, are shown underlined and in bold font. **D** P-value calculated by means of the Fisher’s exact test. P-values comparing different 365 nm radiation time periods. **E** P-value calculated by means of the Fisher’s exact test. P-values comparing different 405 nm radiation time periods.

**Fig 6:**
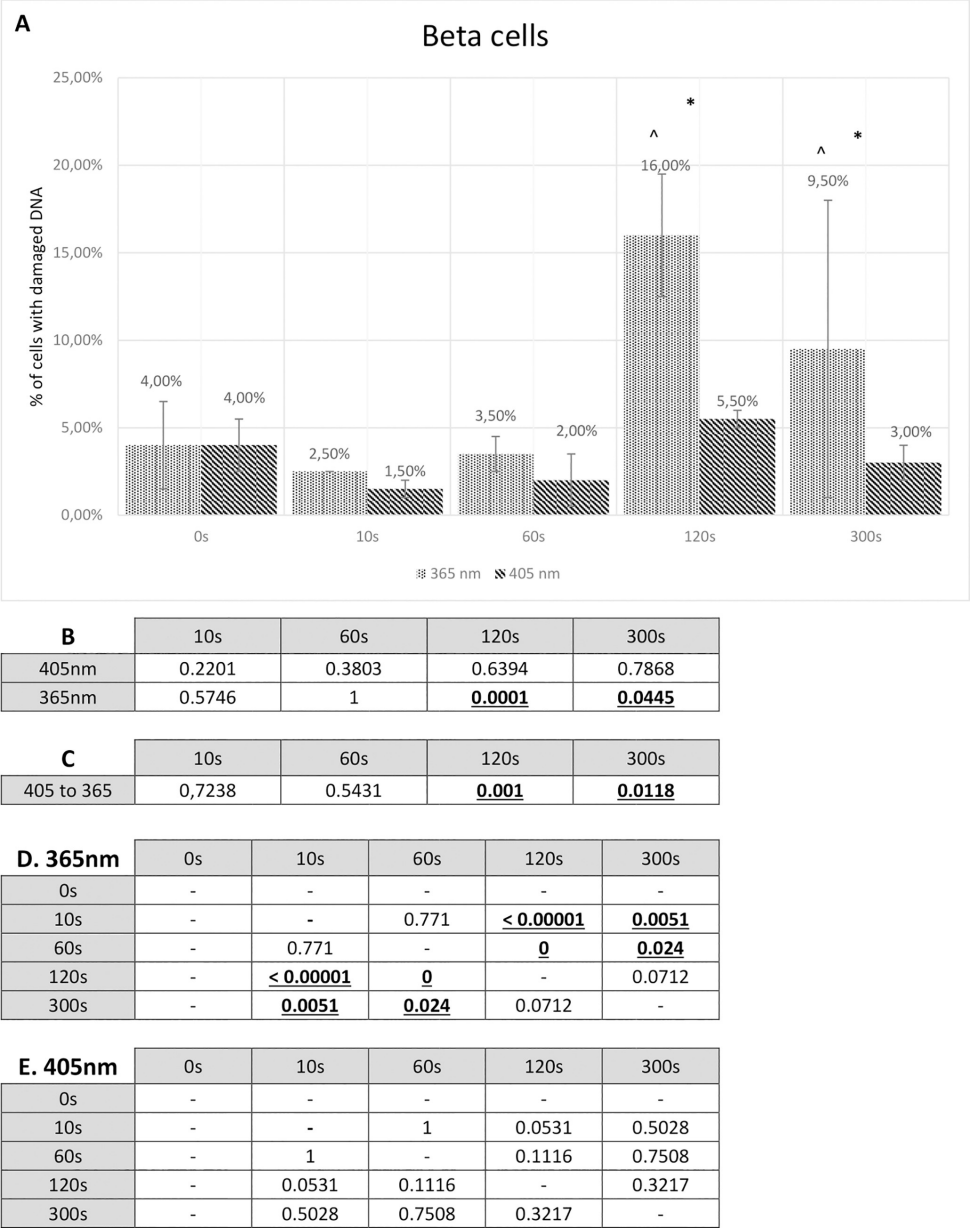
Comet assay results for beta cells. **A** Comet assay results presenting the percentage of beta cells with any degree of DNA damage. The Fisher exact test statistic value comparing 405 nm to 365 nm: * - p < 0.05; ** - p < 0.0001. The Fisher’s exact test statistic value comparing radiation (405 nm and 365 nm) to control: ^ - p < 0.05. Standard deviation bars are present for every value. **B** P-value calculated by means of the Fisher’s exact test. P-value of beta cells used in the comet assay study compared to control. **C** P-value calculated by means of the Fisher’s exact test. P-value of beta cells treated with 405 nm radiation compared to 365 nm radiation. Statistically relevant p-values, where p < 0.05, are shown underlined and in bold font. **D** P-value calculated by means of the Fisher’s exact test. P-values comparing different 365 nm radiation time periods. **E** P-value calculated by means of the Fisher’s exact test. P-values comparing different 405 nm radiation time periods.

Cultured beta cells were shown to be a bit more susceptible to visible blue light and UV light radiation than cultured alpha cells. Radiation using 405 nm wavelength for 10 seconds resulted in 1.5% damage and 365 nm wavelength caused 2.5% damage. Treating beta cells with both wavelengths for 300 seconds gave rise to 3% and 9.5% damage, respectively. Complete results showing the percentage of cultured alpha and beta cells with damaged DNA are presented in the Figs 5A and 6A.

The statistical analysis (by means of the Fisher exact test) which compared both cell lines’ 405 nm radiation to 365 nm radiation showed some level of significance. Alpha cells reached statistically relevant value with 300 seconds of radiation (P-value = 0.0165), while beta cells achieved P-value lower than 0.05 with 120 seconds and 300 seconds (respectively, P-value = 0.001; P-value = 0.0118). These results are presented, for alpha cells in the Fig 5A and 5C, for beta cells in the Fig 6A and 6C. When both wavelengths were set together with a control, radiation with 405 nm was non-relevant in all time periods, either in alpha cells or beta cells. This statistical analysis for alpha and beta cells is shown in the Figs 5B and 6B. Correlation between different periods of both radiation types, for alpha and beta cells, is presented in Figs 5D and 5E and 6D and 6E, respectively.

When it goes to tail intensity measurements of cultured cells, both alpha and beta cells showed low-to-medium tail intensity after treating with 365 nm UV light wavelength. Even 300 s of non-stop UV light resulted in only medium intensity of tails. Table 3 shows tail intensity estimation of 365 nm-treated alpha and beta cells.

### Fibroblasts

**Fig 7:**
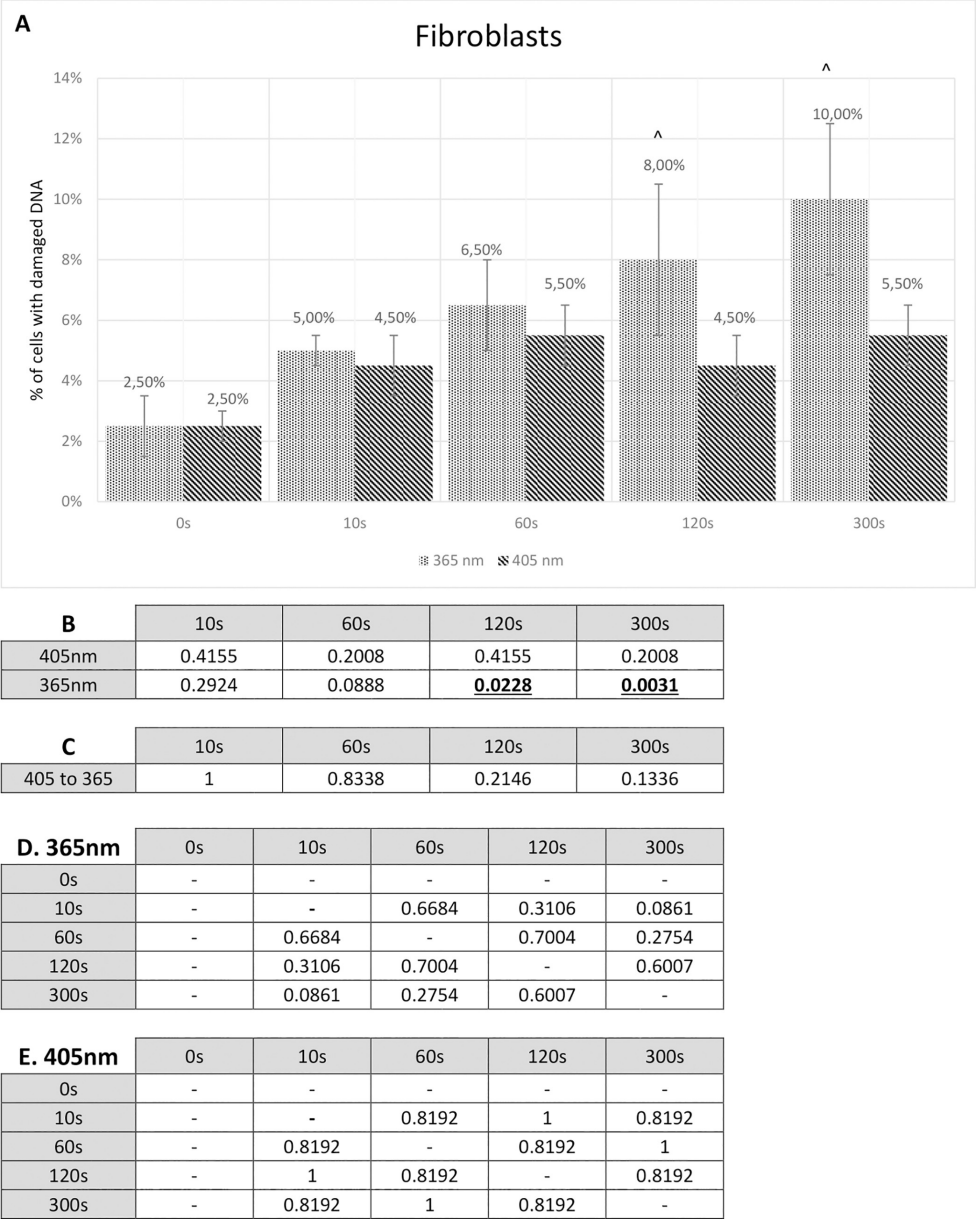
Comet assay results for fibroblasts. **A** Comet assay results presenting the percentage of fibroblasts with any degree of DNA damage. The Fisher exact test statistic value comparing 405 nm to 365 nm: * - p < 0.05; ** - p < 0.0001. The Fisher’s exact test statistic value comparing radiation (405 nm and 365 nm) to control: ^ - p < 0.05. Standard deviation bars are present for every value. **B** P-value calculated by means of the Fisher’s exact test. P-value of fibroblasts used in the comet assay study compared to control. **C** P-value calculated by means of the Fisher’s exact test. P-value of fibroblasts treated with 405 nm radiation compared to 365 nm radiation. Statistically relevant p-values, where p < 0.05, are shown underlined and in bold font. **D** P-value calculated by means of the Fisher’s exact test. P-values comparing different 365 nm radiation time periods. **E** P-value calculated by means of the Fisher’s exact test. P-values comparing different 405 nm radiation time periods.

Human fibroblasts used in this study showed quite similar results to two other cultured cell lines (alpha and beta cells), though on the higher end. Treating fibroblasts with 405 nm wavelength for 10 seconds resulted in 4.5% cells with damaged DNA, whereas 300 seconds of visible blue light radiation caused damage only to 5.5% cells. No significant differences between time periods were shown while 405 nm wavelength radiation. Moreover, cells used as a control (without any radiation treatment) presented 2.5%. Together with the rise of radiation time using wavelength 365 nm, a consistent increase in cells with damaged DNA was observed. 10 seconds of fibroblasts radiation resulted in 5% damage of cell’s genetic material, 120 seconds caused damage to 8% of fibroblasts, while 300 seconds of UV light treatment gave 10% of cells with damaged DNA. What is interesting, distinction between samples treated with 405 nm wavelength and 365 nm wavelength is similar to both alpha cells and beta cells. Complete results showing the percentage of fibroblasts with damaged DNA are presented in the Fig 7A.

The statistical analysis (by means of the Fisher exact test) was used here to compare fibroblast radiation using 405 nm wavelength to 365 nm. These calculations were statistically non-relevant in all time periods, with the lowest P-value reaching 0.1336 (300 seconds). These results are presented in the Fig 7A and 7C. A comparison between both radiation types and a control was also performed. Non-relevant results were presented for all samples with 405 nm radiation, while in two samples with 365 nm radiation (120 seconds and 300 seconds) P-value exceeded under 0.05 (respectively, P-value = 0.0228; P-value = 0.0031). This analysis is shown in the Fig 7B. Additionally, analysis was executed for different time periods of radiation and it showed no statistical significance. It is presented in the Fig 7D and 7E.

Tail intensity visual measurements of fibroblasts. Comets presented after the comet assay were in the category from low to low-to-medium when treated with 365 nm wavelength. 10 seconds of UV light treatment resulted in low intensity of tails. Moreover, after 300 seconds of radiation fibroblast comets reached only low-to-medium tail intensity. Table 3 shows tail intensity estimation of 365 nm-treated fibroblasts.

## Discussion

The results of this study are the first glimpse into UV-induced and visible blue light-induced DNA damage in pancreatic islet cells. No other research papers were found that took into consideration cells of the pancreas. What is more, this is the first publication concentrating solely on the subject of crosslinking with blue visible light and UV light during 3D bioprinting process and its probable consequences considering genetic material of cells embedded in the bioprinted material. Still, there are some patents and articles in which inventors used and studied the visible blue light and UV light crosslinking method in bioprinting [15, 16].

Nevertheless, some studies focused on the impact of UV light on other types of cells. A group of scientists from the Queen’s University in Northern Ireland studied retinal pigment epithelial cells [17]. Their goal was examining qualitative and quantitative features of radiation-induced (by UV-A and UV-B) DNA damage in those cells. As in this currently presented research, they implemented the comet assay because this technique is found to be a very efficient way of gathering information about DNA damage and even its types. It serves a broad range of prospects, from measuring the amount of double-strand DNA breaks, through enabling detection of oxidized pyrimidines, to exposure of UV-induced cyclobutene pyrimidine dimers (CPD) [18].

Human fibroblasts used in our research served more as a control group since a study considering DNA damage in human fibroblasts was published in the past by Rosenstein’s group [19]. They performed an analysis of DNA damage induction through a range (large spectrum) of wavelengths, with 365 nm and 405 nm also being ones studied. Still, they focused only on inducing single-strand DNA breaks and prevented the possibility of DNA repair.

The reason for our experimentation was assessing whether visible blue light-dependent or UV-dependent crosslinking during 3D bioprinting process can cause any damage to cells and micro-organs. It is extremely important to examine all the possible faults of cells that undergo bioprinting, since in the future structures it produces will be transplanted into patients in need of organs. The group from Queen’s University prolonged radiation time in their experiments and they reached higher DNA damage. Similar experimentations were performed by Claudia Bock’s scientists from Germany [20]. To mimic the crosslinking procedure during bioprinting process, the period of radiation didn’t exceed 5 minutes in any of the studied material in our experiments. 3D bioprinted prototypes of tissues and organs are not being printed in large size, since they need to resemble their natural equivalents only in functionality, not in size (they also need to fit into a 3D bioprinter). Hence, even when we assume crosslinking agent being implemented after each and every bioprinted layer, the overall time of direct contact of visible blue light or UV light will hardly ever surpass given 5 minutes [21].

Until quite recently, most of the focus was given to research about the impact of UV light on DNA. Together with the rise of tissue and organ bioengineering, the visible blue light started to attract scientists in search of an ideal sterilisation method of biomaterials. Visible blue light has been previously reported to be used in bacterial decontamination. What is more, for this reason it gained popularity as a skin treatment agent (for example treatment of acne) [22]. Comparatively with the results from the University of Strathclyde (Glasgow, UK) considering murine fibroblasts and their viability [23], our results suggest that visible blue light (405 nm wavelength) has no noticeable effect on either alpha and beta cells, fibroblasts or isolated pancreatic islets. Furthermore, the percentage of cells with damaged DNA in all studied samples was as low as in the control group [Figs 3A, 5A, 6A, 7A], showing similarities with another study from Radboud University Nijmegen Medical Centre where they took a closer look at the effects of visible blue light on healthy human skin [24]. All these results together are in contradiction to some studies reporting that visible light (especially near-UV wavelength) may cause DNA damage [25]. Moreover, statistical analysis of our results (P-value by means of the Fisher exact test) considering all studied cell lines and all time periods suggests that the difference between 405 nm radiation and UV light radiation is statistically relevant [Figs 3C, 5C, 6C, 7C]. It is also confirmed by the separate analysis of visible blue light radiation in comparison to controls, where all calculations proved to be statistically non-relevant, which means that a distinction between control and all time periods was very low [Figs 3B, 5B, 6B, 7B]. The only statistical relevance that may cause some uncertainty is pancreatic islets radiation with 405 nm, where two of calculations comparing visible blue light radiation with a control were statistically relevant (P-value lower than 0.05 but higher than 0.01) [Fig 3B]. Still, the difference between both radiation types was significant and percentage of cells with damaged DNA after 405 nm radiation was very low in contradiction to UV light radiation. This can be explained by the fact that pancreatic islets after isolation are more fragile and more susceptible to external damaging agent, especially when there is no exocrine tissue sheltering them. Pancreatic islets as a biological material have very diverse characteristics, depending on many variables such as the quality of an organ procured, isolation procedure and culturing conditions. As mentioned previously, it is possible that significantly longer radiation period in this study could have a stronger impact on DNA and therefore show more DNA damage caused even by visible blue light. One 405 nm radiation period (90 seconds) turned out to be statistically significant, thus proving once again the variety of biological material. Based on this it can be proposed that using 405 nm radiation on pancreatic islets is safe for the first 60 seconds, which is a sufficient time for the crosslinking process of bioprinted materials. Radiation for longer time (90 seconds and longer) gives variable results. Even though it causes significantly lower damage than 365 nm, the safety of its use remains uncertain and needs further analysis [Fig 3B–3E].

Surprisingly, the main difference while analysing comet assay results was observed between samples with isolated pancreatic islets and cultured cell lines. Pancreatic human islets are composed from the following types of endocrine cells: alpha (~25%), beta (~60%), delta (~10%) and a few PP-cells. Porcine pancreatic islets have a similar composition, though with a higher amount of beta cells, which are mostly present in the core of an islet [26, 27]. Theoretically then, the radiation should affect it in a very similar way to its interaction with cultured alpha and beta cells. Nonetheless, UV radiation alone resulted in a much higher overall percentage of cells with damaged DNA, while treating pancreatic islets with visible blue light didn’t cause any noticeable dissimilarities compared to cultured cell lines. It could be argued that the reason for this is the method of pancreatic islet isolation itself. Pancreatic islet isolation, whether human or swine, is an extremely complex process, with many variables impacting the final result–the quantity and quality of pancreatic islets. The use of collagenase and protease, along with many other reagents, not only helps to free pancreatic islets from the pancreas exocrine scaffold. It can also have some impact on the strength of those pancreatic islets [28,29]. It has not yet been studied whether the isolation process influences the DNA integrity of pancreas. What is more, pancreatic islets after isolation are not cultured as it is the case with all other biological material. They act as mini-organs and that is how scientists and medics treat them. They only stay in specially designed media in petri dishes or culture bottles to ensure their survival till experimentation or transplantation takes place. Hence, the time of experimentation after the isolation process could be a significant factor in the UV radiation studies, since it can impact the DNA integrity of exocrine cells of pancreatic islets.

## Conclusion

Radiation with 365 nm wavelength (UV light) is not suitable for live cells because it results in DNA damage. Therefore, a novel type of radiation was introduced among various fields of science–visible blue light (405 nm). Many studies proved that it is comparable to that of 365 nm wavelength but it is far less harmful to DNA of cells. In this study we compared the impact of both wavelengths on swine pancreatic islets, α-cells, β-cells and fibroblasts. It confirmed previous findings, radiation using 405 nm wavelength generates a much smaller amount of DNA damage in the studied biological material. This uncovers various possibilities for using this type of radiation, an example being crosslinking with visible blue light during the process of 3D bioprinting. It is common to use 365 nm photo-crosslinking of biomaterials in this process. However, our study proves its harmfulness while implementing live cells for 3D bioprinting. For that reason we experimented with 405 nm wavelength in terms of its impact on the attempt to 3D bioprint a bionic pancreas (using pancreatic islets and cell lines mentioned above) This study is a preliminary study performed without bioinks, since we focused on the comet assay. Hence, further research needs to be performed assessing the DNA damage of cells embedded in various bioinks. 3D bioprinting of porcine islets and a whole bionic pancreas is still being developed. Thus, examining all individual steps of this process will allow us to create a technology that can be safely moved to clinics in the future.
